# The Role of Oxidative Stress in Decreased Acetylcholinesterase Activity at the Neuromuscular Junction of the Diaphragm during Sepsis

**DOI:** 10.1155/2017/9718615

**Published:** 2017-11-05

**Authors:** Hua Liu, Jin Wu, Jun-yan Yao, Hong Wang, Shi-tong Li

**Affiliations:** ^1^Department of Anesthesiology, Shanghai General Hospital, Shanghai Jiao Tong University School of Medicine, Shanghai 200080, China; ^2^Department of Anesthesiology, The Ninth People's Hospital, Shanghai Jiao Tong University School of Medicine, Shanghai 200011, China

## Abstract

Our recent study demonstrated that acetylcholinesterase (AChE) activity at the neuromuscular junction (NMJ) of the diaphragm decreased during sepsis. However, the mechanisms were not clearly identified. In this study, we aimed to investigate whether the decreased AChE activity was related to oxidative stress by observing AChE activity in different grades of sepsis induced by caecal ligation and puncture (CLP). At 24 h after surgery, an assay of thiobarbituric acid reactive species (TBARS) and protein carbonyls, as well as the myeloperoxidase (MPO), superoxide dismutase (SOD), and catalase (CAT) activity, was conducted. AChE activity was measured by biochemical and histological detection. AChE and CAT activity in the diaphragm decreased, while the contents of TBARS and protein carbonyls, the activity of MPO and SOD, and the SOD/CAT ratios increased. The above changes were much more significant in the mid-grade septic group than in the low-grade septic group. The colour of the AChE activity staining at the NMJ gradually lightened from the sham surgery group to the mid-grade septic group. AChE activity was significantly negatively correlated with the levels of TBARS and protein carbonyls. We consider that oxidative stress might be responsible for decreased AChE activity in the diaphragms of rats induced with sepsis.

## 1. Introduction

Sepsis is a clinical syndrome caused by severe infection, leading to multiple organ dysfunction [1, 2]. Our recent study has reported that acetylcholinesterase (AChE) activity at the neuromuscular junction (NMJ) of the diaphragm was inhibited during sepsis [3]. However, the effects of different grades of sepsis on the activity of AChE were not illustrated in that study. Moreover, the mechanisms through which AChE activity was inhibited during sepsis were not clearly identified.

Previous studies have demonstrated that several factors, including inflammatory, immune, hormonal, metabolic, and bioenergetic responses, were involved in the pathogenesis of sepsis [4–6]. One of the crucial factors in these processes is the loss of balance between the reactive species (ROS) and antioxidant systems, leading to a state of irreversible oxidative stress [7]. ROS contain a series of active oxygen free radical or molecule, including superoxide (O_2_^•−^), hydrogen peroxide (H_2_O_2_), hydroxyl radicals (^•^OH), and hypochlorous acid (HOCl) [8, 9], which can attack proteins, carbohydrates, nucleic acids, and unsaturated lipids, subsequently causing tissue and organism injury [10]. Several previous studies have found that ROS can inhibit AChE in various tissues [11–17].

These findings raise the possibility that oxidative stress might be a vital contributor to the inhibition of AChE activity during sepsis. In the current study, we detected AChE activity in two different grades of sepsis to more intensively determine the effects of sepsis on AChE activity. Meanwhile, we measured the indicators of oxidative injury (thiobarbituric acid reactive species (TBARS) and protein carbonyl) to observe the correlation between AChE activity and oxidative stress during sepsis. We aimed to clarify the role of oxidative stress in the decreased AChE activity at the NMJ of diaphragm during sepsis.

## 2. Materials and Methods

### 2.1. Animals and Experimental Protocol

Our study was approved by the Animal Care and Use Committee of Shanghai General Hospital Affiliated to Shanghai Jiao Tong University (Grant number 2017DW001). Experiments were performed using a total of 28 adult male Sprague-Dawley rats weighing between 220 and 260 g. The animals were allowed unrestrained access to food and water and housed at an ambient temperature of 23–25°C with 12 h light-dark cycles throughout the study. The rats were randomly divided into three groups: (1) the sham group (S group, *n* = 8), (2) the low-grade sepsis group (L group, *n* = 8), and (3) the midgrade sepsis group (M group, *n* = 12 due to an expected mortality rate of approximately 25% within the first 24 h in our previous study). The rats were anaesthetized with intraperitoneal injection of pentobarbital (50 mg/kg). In the L and M groups, sepsis was surgically induced using the CLP method, as it is widely used and known to closely mimic the pathophysiology of septic patients [18]. Under aseptic conditions, a 3 cm midline abdominal incision was performed to expose the caecum, with adjoining intestine. The caecum in the L group was ligated near the distal pole comprising 10% of the caecum, whereas the caecum in the M group was ligated in the middle portion [19]. Then, the caecum was perforated once with an 18-gauge needle. Faecal droplets were squeezed out of the caecum through penetration holes. The caecum was then relocated into the abdominal cavity, and the laparotomy was closed with 3-0 silk sutures. In the S group, a midline abdominal incision was made, and the caecum was exposed but not ligated or perforated. All rats were immediately resuscitated with prewarmed (37°C) normal saline (50 ml/kg subcutaneous). All animals were allowed to return to their cages, with free access to food and water after surgery.

At 24 h after surgery, the surviving rats were euthanized through intraperitoneal injection of pentobarbital (100 mg/kg), and their diaphragms were harvested. The right hemidiaphragm was removed and immediately stored at −80°C until being assayed for AChE activity, as well as the contents of TBARS formation and protein carbonyls, and MPO, SOD, and CAT activities. The ventral costal region of the left hemidiaphragm was immediately removed for histological detection of AChE activity at the NMJ.

### 2.2. Histological Detection of AChE Activity at the NMJ

The AChE activity at the NMJ of the diaphragm was detected through a modified Karnovsky and Roots method by referencing our previous study [3]. The brown insoluble products on the muscle fibres indicated AChE activity [20].

### 2.3. Biochemical Measurement of AChE Activity

The method is based on the fact that AChE can hydrolyse acetylcholine to form acetic acid and choline. The latter can react with a mercapto colour reagent to form a yellow symmetrical trinitrobenzene compound. Measuring the colour depth of the compound can reflect the activity of AChE. The AChE activity assay test was carried out using an AChE assay kit from the Nanjing Jiancheng Bioengineering Institute (A024, Nanjing, China) according to the manufacturer's instructions [21]. The results were determined by measuring the absorbance at 412 nm and expressed as units of AChE activity per milligram of protein.

### 2.4. TBARS Measurement

As an indicator of lipid peroxidation in oxidative stress, we determined the formation of TBARS during an acid-heating reaction, as previously described [22]. Briefly, the samples were mixed with 1 ml of trichloroacetic acid 10% purchased from Sigma Chemical (St. Louis, MO, USA) and 1 ml thiobarbituric acid 0.67%, and then, the mixture was heated in a boiling water bath for 15 mins. TBARS was determined by measuring the absorbance at 535 nm, and the results are expressed as malondialdehyde (MDA) equivalents per milligram of protein.

### 2.5. Measurement of Protein Carbonyls

As a marker of the oxidative damage to proteins, protein carbonyl groups were measured based on the reaction with dinitrophenyl hydrazine, as previously described [23]. Briefly, the proteins were precipitated by adding 20% trichloroacetic acid and redissolved in dinitrophenyl hydrazine. Eventually, the protein carbonyls were determined by reading the absorbance at 375 nm, and the results were expressed as nmols of protein carbonyls per milligram of protein.

### 2.6. MPO Activity Assay

MPO activity in homogenates of diaphragm tissue was determined using a test kit from Nanjing Jiancheng Bioengineering Institute (A044, Nanjing, China). Activity was measured spectrophotometrically as the change in absorbance at 412 nm, using a Spectramax microplate reader. The results are expressed as units of MPO activity per gram of tissue wet weight.

### 2.7. Measurement of CAT and SOD Activities

The above-prepared supernatant was used for CAT and SOD activity assay according to the instructions of their corresponding test kit purchased from Nanjing Jiancheng Bioengineering Institute (CAT: A007-1; SOD: A001-1, Nanjing, China). Both results were expressed as units of enzyme activity per gram of tissue wet weight. Finally, the ratio of SOD/CAT was calculated and recorded.

### 2.8. Statistical Analyses

SPSS (version 19.0; SPSS Inc., Chicago, Illinois, USA) was used for the statistical analysis. Values are expressed as the mean ± standard deviation (SD). The data were tested for normality and equality of variance. Between-group comparisons for each dependent variable were assessed using analysis of variance (ANOVA) with the least significant difference (LSD) test. Pearson's correlation analysis was used to determine the association between AChE activity and TBARS or protein carbonyls, and *P* < 0.05 was considered to be statistically significant.

## 3. Results

### 3.1. Mortality within 24 h

Four of the 12 rats in the M group died within 24 h, and all other rats survived during the first 24 h. In addition, the rats in midgrade septic group developed more severe septic symptoms, such as shortness of breath, hair erection, subconjunctival haemorrhage, diarrhoea, and loss of movement, than those in low-grade septic group.

### 3.2. AChE Activity Staining at the NMJ

**Figure 1:**
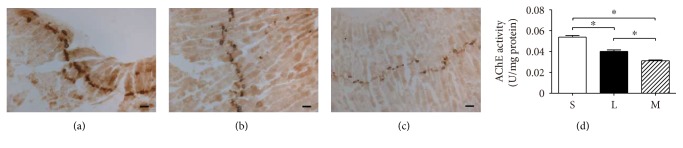
AChE staining at neuromuscular junction in sham group (a), low-grade septic group (b), and midgrade septic group (c). Bars = 50 *μ*m. Comparison of AChE activity through biochemical measurement in three groups of rat diaphragm (d). The values are expressed as means ± SD. *n* = 8, ^∗^*P* < 0.01. S: sham group; L: low-grade septic group; M: midgrade septic group; SD: standard deviation.

As shown in Figure 1, AChE staining was detected at motor endplate regions in rat diaphragms from all three groups. Observed from the slices, the brown insoluble products of AChE staining in the S group were deepest, while the brown in M group was lightest (Figures 1(a), 1(b), and 1(c)).

### 3.3. AChE Activity in the Diaphragm

Compared with the S group, AChE activity decreased significantly in both the L and M groups (*P* < 0.01). Furthermore, the decline of AChE activity in the M group was much more significant than that in the L group (*P* < 0.01) (Figure 1(d)).

### 3.4. Oxidative Parameters at the Diaphragm

#### 3.4.1. TBARS and Protein Carbonyls in the Diaphragm

**Figure 2:**
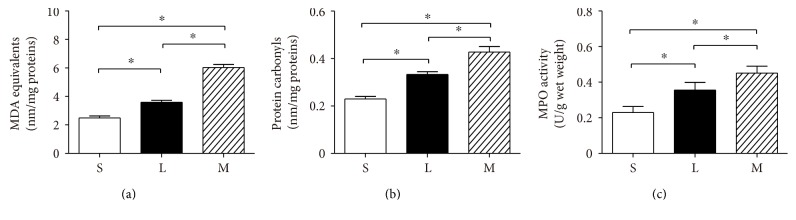
Comparison of thiobarbituric acid reactive species (a), protein carbonyls (b), and MPO activity (c) in three groups of rat diaphragm. The values are expressed as means ± SD. *n* = 8, ^∗^*P* < 0.01. S: sham group; L: low-grade septic group; M: midgrade septic group; SD: standard deviation.

Twenty-four hours after CLP (in both the L and M groups), TBARS and protein carbonyls increased significantly in the diaphragm (*P* < 0.01). Additionally, the levels of TBARS and protein carbonyls were higher in the M group than those in the L group (*P* < 0.01) (Figures 2(a) and 2(b)).

#### 3.4.2. MPO Activity in the Diaphragm

As illustrated in Figure 2(c), MPO activity was significantly elevated in rats in both the L and M groups compared to rats in the S group (*P* < 0.01). Moreover, MPO activity was higher in the M group than that in the L group (*P* < 0.01) (Figure 2(c)).

### 3.5. Antioxidant Enzyme Activities

#### 3.5.1. SOD and CAT Activities and SOD/CAT Ratios

**Figure 3:**
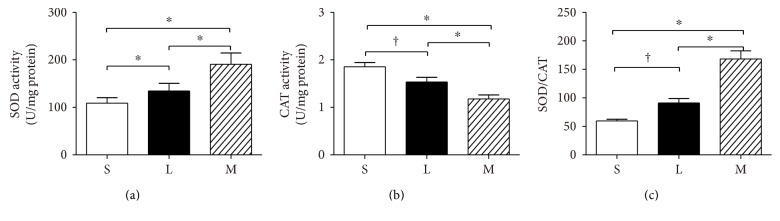
Comparison of SOD activity (a), CAT activity (b), and SOD/CAT (c) in three groups of rat diaphragms. The values are expressed as the means ± SD. *n* = 8, ^∗^*P* < 0.01, ^†^*P* < 0.05. S: sham group; L: low-grade septic group; M: midgrade septic group; SD: standard deviation.

SOD activity increased in both the L and M groups (*P* < 0.01), and the increase of SOD activity was higher in the M group than that in the L group (Figure 3(a)). In contrast, a significant decrease was observed in CAT activity in the L (*P* < 0.05) and M groups (*P* < 0.01). CAT activity decreased more in the M group than in the L group (Figure 3(b)). The ratio of SOD and CAT increased significantly in the L group (*P* < 0.05) and M group (*P* < 0.01). Meanwhile, this ratio was higher in the M group than that in the L group (*P* < 0.01) (Figure 3(c)).

### 3.6. Correlation between AChE Activity and TBARS or Protein Carbonyls

**Figure 4:**
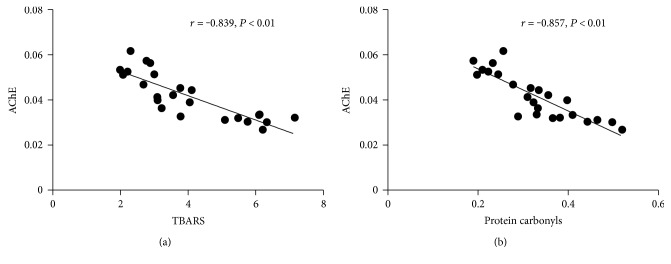
Correlation between AChE activity and TBARS (a) or protein carbonyls (b).

AChE activity was significantly and negatively correlated with the levels of TBARS (*r* = −0.839, *P* < 0.01) (Figure 4(a)) and protein carbonyls (*r* = −0.857, *P* < 0.01) (Figure 4(b)).

## 4. Discussion

Using low-grade and midgrade models of sepsis in rats, this study found that the AChE and CAT activities in the diaphragm decreased, while the contents of TBARS and protein carbonyls, the activity of MPO and SOD, and the SOD/CAT ratios increased at 24 h after CLP surgery. Furthermore, the above changes were much more significant in the midgrade septic group than those in the low-grade septic group. Strikingly, we found that AChE activity was significantly and negatively correlated with the levels of TBARS and protein carbonyls in rats.

In this study, low-grade and midgrade sepsis was established by ligating different caecum lengths, as described in previous studies [19]. The rats in the midgrade septic group had a higher mortality rate and developed more severe septic symptoms than those in the low-grade septic group. Moreover, TBARS and protein carbonyls, which represent the lipid and protein injury, respectively, in oxidative stress increased more in the midgrade septic group than in the low-grade septic group. These results illustrated that our sepsis models of different severity grades were successfully created.

AChE activity was found to be significantly and negatively correlated with the levels of TBARS and protein carbonyls, which indicated that oxidative stress may contribute to the decreased AChE activity during sepsis. It is believed that ROS plays an important role in the oxidative stress to cause cellular injury during sepsis. ROS in the diaphragm may primarily be derived from mitochondrial respiration chain impairment and infiltrating inflammatory cells [24, 25]. O_2_^•−^ produced through the above two manners can be converted to H_2_O_2_ by SOD. H_2_O_2_ will be converted to harmless H_2_O and O_2_ through the action of CAT. Otherwise, it will allow neutrophils to oxidize chloride ions into HOCl through MPO [26]. SOD and CAT are two oxidative enzymes that play vital roles in eliminating ROS. Some research has revealed that SOD activity increased without a proportional increase in CAT activity during sepsis [27, 28]. In our study, the imbalance also existed, and it was much more serious in midgrade sepsis than in low-grade sepsis. The different modulation of SOD and CAT during sepsis may rise from some reasons below. SOD activity increased in the CLP surgery probably as a response to oxidative stress induced by sepsis. SOD activation during sepsis could be a compensatory response to the overproduction of mitochondrial O_2_^•−^ so as to eliminate excessive O_2_^•−^[28]. Additionally, the increase of interleukin-1 (IL-1), tumor necrosis factor (TNF), and lipopolysaccharide (LPS) during sepsis could also activate SOD [29, 30]. Unlike SOD, the previous study demonstrated that excessive O_2_^•−^ or H_2_O_2_ could oxidize CAT active site leading to enzymatic inactivation [31]. The imbalance between SOD and CAT may result in the accumulation of excessive H_2_O_2_ in the diaphragm [27, 32]. Additionally, our results indicated that MPO activity increased in both septic groups, and the activity increased higher in midgrade sepsis than in low-grade sepsis. MPO, synthesized and secreted by neutrophils, is a marker of inflammation and neutrophil infiltration in tissues [33]. As described above, increased MPO can further convert excessive H_2_O_2_ into HOCl. Moreover, excessive H_2_O_2_ will react with iron to generate ^•^OH through Fenton chemistry [28].

Early in 1966, O'Malley et al. found that both H_2_O_2_ and peroxides could inhibit erythrocyte AChE [11]. A study from Danylovych reported that AChE of myometrium sarcolemma could be inhibited by H_2_O_2_ [12]. Accumulation of H_2_O_2_ in the mM range can decrease epidermal AChE expression and inhibit human recombinant AChE activity [13, 14]. Additionally, it has been verified that ^•^OH inhibits rat brain AChE and human recombinant AChE activity [15, 16]. In addition, HOCl has been found to be a strong inhibitor of AChE [17]. A kinetic analysis using pure recombinant human AChE and a molecular modelling based on the established 3D structure of human AChE supported that ROS-mediated oxidation of Trp432, Trp435, and Met436 moves and disorients the active site His440 of AChE, leading to deactivation of the protein [13, 14]. Moreover, TBARS can decrease membrane fluidity and decrease AChE activity through lipid-protein interactions [15]. These results provided a molecular basis through which oxidative stress inhibits AChE activity during sepsis.

However, our study has two limitations. First, we failed to observe the conformation change of AChE induced by ROS directly because of the limitations of the experimental methods. Second, we did not detect the quantity of AChE expression simultaneously, although recent data from another study in our laboratory revealed that such data may be another factor affecting the decrease of AChE activity (unpublished data).

## 5. Conclusions

We believe that there were at least two highlights in the present study. First, we found that AChE activity at the NMJ of diaphragm decreased more significantly during severe sepsis for the first time. Second, AChE activity at the NMJ of diaphragm is found to be significantly and negatively correlated with level of oxidative stress during sepsis.

In conclusion, our study verified that oxidative stress might be responsible for the decreased activity of the AChE at the NMJ in the diaphragm. Furthermore, the effects of antioxidant measurements on AChE activity during sepsis should be investigated in the future.
